# Evaluation of super-resolution deep learning reconstruction on three-dimensional constructive interference in steady state for enhanced visualization of vestibular schwannomas

**DOI:** 10.1007/s12194-026-01073-7

**Published:** 2026-06-01

**Authors:** Akira Katayama, Koichiro Yasaka, Miori Fujimoto, Yusuke Asari, Satoru Kamio, Jun Kanzawa, Yuki Sonoda, Shigeru Kiryu, Osamu Abe

**Affiliations:** 1 Department of Radiology, The University of Tokyo, Tokyo, Japan; 2 Department of Radiology, International University of Health and Welfare, Chiba, Japan

**Keywords:** Magnetic resonance, Super-resolution deep learning reconstruction, Vestibular schwannoma, MR cisternography, Cerebellopontine angle, Vestibulocochlear nerve

## Abstract

We evaluated whether, compared with conventional deep learning reconstruction (DLR) and zero-filling interpolation (ZIP), super-resolution DLR (SR-DLR) enhances the visualization of vestibular schwannomas and cranial nerves in three-dimensional constructive interference in steady state (3D-CISS) magnetic resonance (MR) imaging. This retrospective study analyzed 39 patients with vestibular schwannomas who underwent 3T MR imaging with 3D-CISS. Axial images were reconstructed using SR-DLR, DLR, and ZIP. Three readers independently evaluated tumor depiction, depiction of facial and vestibulocochlear nerves, sharpness, noise, artifacts, and diagnostic acceptability. Quantitative indicators included edge rise distance (ERD) and edge rise slope (ERS) measured along a linear region of interest (ROI) drawn perpendicularly across the tumor–cistern cerebrospinal fluid (CSF) boundary, and signal-to-noise ratio (SNR), contrast-to-noise ratio (CNR) and contrast ratio (CR) calculated from circular ROIs (3–5 mm) placed in the tumor and prepontine CSF. SR-DLR significantly improved visualization of vestibular schwannoma and facial and vestibulocochlear nerves compared with ZIP (*p* < 0.001). Nerve depiction ratings varied between readers when comparing SR-DLR and DLR. SR-DLR yielded significantly better sharpness, lower ERD and higher ERS than both DLR and ZIP (*p* < 0.001). Artifacts showed no significant differences. No clear deterioration in noise was noted. Objectively, SR-DLR showed significantly higher CR than DLR (*p* = 0.023) and ZIP (*p* < 0.001). SNR and CNR were significantly higher with SR-DLR than ZIP, but comparable to DLR. SR-DLR improves sharpness and lesion conspicuity on 3D-CISS imaging without a clear increase in artifacts or noise compared with ZIP.

## Introduction

Vestibular schwannomas, also known as acoustic neuromas, are benign tumors originating from the Schwann cells of the vestibular nerve, which typically arise in the cerebellopontine angle (CPA) and the internal auditory canal [1]. They account for approximately 10% of all intracranial tumors, and their incidence is estimated to be between 10 and 20 cases per million population annually [2–4]. Approximately 30–90% of vestibular schwannomas exhibit growth, although the rate is usually slow, at less than 2 mm per year [5, 6]. Currently, the utility of the three-dimensional constructive interference in steady state (3D-CISS) in magnetic resonance (MR) imaging has been recognized for evaluating these small tumors located in the CPA [7–9]. Images with high spatial resolution are therefore essential for delineating them. It is generally known that reducing voxel size to improve image quality decreases the signal per voxel and increases noise. To that end, zero-filling interpolation (ZIP), which improves the spatial resolution of reconstructed MR images, is commonly used in routine clinical practice [10, 11]. This method increases the apparent number of pixels and smooths the image. However, ZIP has limitations, producing specific artifacts known as ring artifacts when the interpolation coefficient is high [12].

In recent years, the application of deep learning in the field of radiologic diagnosis has rapidly progressed, demonstrating potential not only for diagnostic support, but also for image processing. Deep learning reconstruction (DLR) has been attracting attention as a technique that reduces image noise and improves image quality. The more recently developed super-resolution DLR (SR-DLR) technique has been reported to improve the spatial resolution of MR images [13]. For example, SR-DLR has been shown to enhance the visualization of cranial nerves in 3D-T2WI-fast asymmetric spin echo images [14]. 3D-CISS imaging is known to be susceptible to banding artifacts, susceptibility artifacts, and motion artifacts [15]. In images with many artifacts, there is concern that DLR may enhance these [16, 17]. On the other hand, SR-DLR has the potential to dramatically improve the visualization of minute structures by achieving both high spatial resolution and low noise. However, to our knowledge, no reports exist evaluating the impact of DLR and SR-DLR on 3D-CISS imaging in the CPA region or assessing their effects on tumor visualization capability. We investigated whether, compared with conventional DLR and ZIP, SR-DLR improves the visualization of vestibular schwannomas and cranial nerves in 3D-CISS imaging.

## Methods

This retrospective study was approved by the Institutional Review Board of our hospital, and the requirement for written informed consent was waived given the retrospective nature of the study.

### Patients

**Fig. 1:**
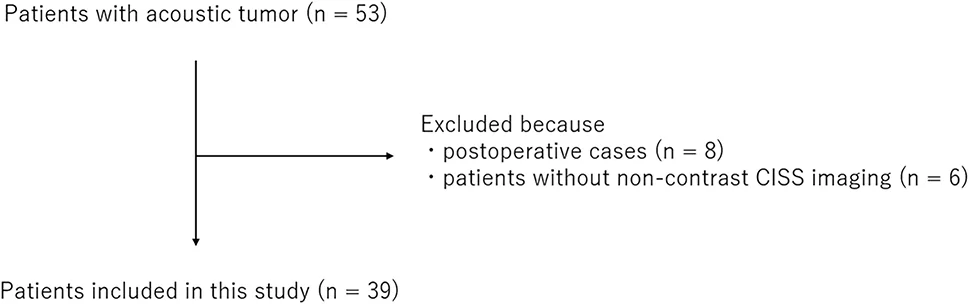
Patient inclusion flowchart. *CISS* constructive interference in steady state

Patients who underwent 3T MR imaging between July 2024 and March 2025 were retrospectively reviewed. Eligible patients had undergone axial 3D-CISS imaging and had been diagnosed with vestibular schwannomas. Of 53 patients initially identified, 14 were excluded based on these criteria: imaging was postoperative (*n* = 8) or lacked a noncontrast 3D-CISS sequence (*n* = 6). Consequently, 39 patients (mean age: 60.1 ± 12.7 years; 13 men, 26 women) were included in the analysis (Fig. 1). The indications for MR imaging were follow-up after gamma knife therapy (*n* = 29), untreated observation of vestibular schwannomas (*n* = 8), and evaluation for vertigo (*n* = 2).

### Acquisition of MR images

MR imaging was performed using a 3T MR unit (Vantage Centurian: Canon Medical Systems, Otawara, Japan). The axial 3D-CISS imaging parameters used were: repetition time, 5.50 ms; echo time, 2.75 ms; flip angle, 50 degrees; echo train length, 1; acquisition matrix, 256 × 240; pixel bandwidth, 488 Hz; and number of excitations, 1.

### Image reconstruction

All reconstruction methods were obtained from the same raw data. Image reconstruction was performed using SR-DLR (Precise IQ Engine: Canon Medical Systems), conventional DLR (Advanced intelligent Clear-IQ Engine: Canon Medical Systems), and ZIP. The reconstruction parameters used were: slice thickness, 0.8 mm for all methods; pixel spacing, 0.1667 mm for SR-DLR and 0.2500 mm for DLR and ZIP; and matrix size, 960 × 960 for SR-DLR and 640 × 640 for DLR and ZIP. The ZIP factor was 3 for SR-DLR and 2 for both DLR and ZIP. For SR-DLR, we used Level 3 Medium, and for DLR, Level 3 Medium Adaptive was applied.

### Principles of the SR-DLR algorithm

The SR-DLR algorithm consists of two main processes. First, as in conventional DLR, noise reduction is performed using a neural network. Second, the processed images undergo fast Fourier transformation followed by zero-filling interpolation in k-space to enhance spatial resolution. Afterward, an inverse fast Fourier transformation is performed, and a second neural network trained to minimize the artifacts introduced by zero-filling is applied. This two-step process results in improved in-plane spatial resolution while image quality is maintained.

### Qualitative image analysis

Before evaluation, all image sets (SR-DLR, DLR, ZIP) were randomized by a board-certified radiologist with 15 years of experience. Three readers (1, 2, and 3, with six years, three years, and one year of radiology experience respectively) used the ImageJ software (https://imagej.net/ij/) to independently evaluate the images. The readers were blinded to patient information and the reconstruction methods. Images from patients who had been excluded from the analysis were used for reader training (familiarization with the scoring system) before the study evaluation began. Each reader independently assessed these categories:

*Tumor depiction (4-point scale)
  (4, excellent; 3, slightly blurred; 2, moderately blurred; 1, poorly visible)
*Cranial nerve depiction (facial and vestibulocochlear nerves; 4-point scale).
  (4, all nerves clearly identifiable; 3, all nerves identifiable but partly blurred; 2, nerves not separated clearly; 1, nerves poorly visible)
*Image sharpness (4-point scale)
  (4, sharp; 3, mild blurring; 2, moderate blurring; 1, severe blurring affecting diagnosis)
*Image noise (4-point scale)
  (4, almost no noise; 3, mild noise; 2, moderate noise; 1, severe noise affecting diagnosis)
*Artifacts (3-point scale); Artifacts were qualitatively evaluated by the readers using a 3-point scale. This score represented an overall assessment of various artifacts, including banding, susceptibility, and motion artifacts commonly encountered in 3D-CISS imaging, as well as ghost artifacts reported to occur with DLR and ringing artifacts that may arise from high ZIP coefficients.
  (3, minimal or no artifacts; 2, recognizable but nondisturbing artifacts; 1, artifacts interfering with diagnosis)
*Diagnostic acceptability (5-point scale).
  (5, excellent; 4, better than standard; 3, standard/acceptable; 2, borderline; 1, unacceptable)

### Quantitative image analysis

A radiology resident with four years of experience used the ImageJ software to perform quantitative image quality analysis under the supervision of a senior radiologist (15 years of experience). Measurements were performed three times by the same examiner, and the average value was used.

These indicators were measured:

*Edge rise distance (ERD)—a shorter ERD indicates greater sharpness.
*Edge rise slope (ERS)—a steeper ERS indicates greater sharpness.
*Signal-to-noise ratio (SNR)—calculated for both the tumor and CSF.
*Contrast-to-noise ratio (CNR)—calculated between the tumor and CSF.
*Signal intensity (SI)—measured for both the tumor and CSF.
*Contrast ratio (CR) —calculated from the signal intensities of the tumor and CSF.

**Fig. 2:**
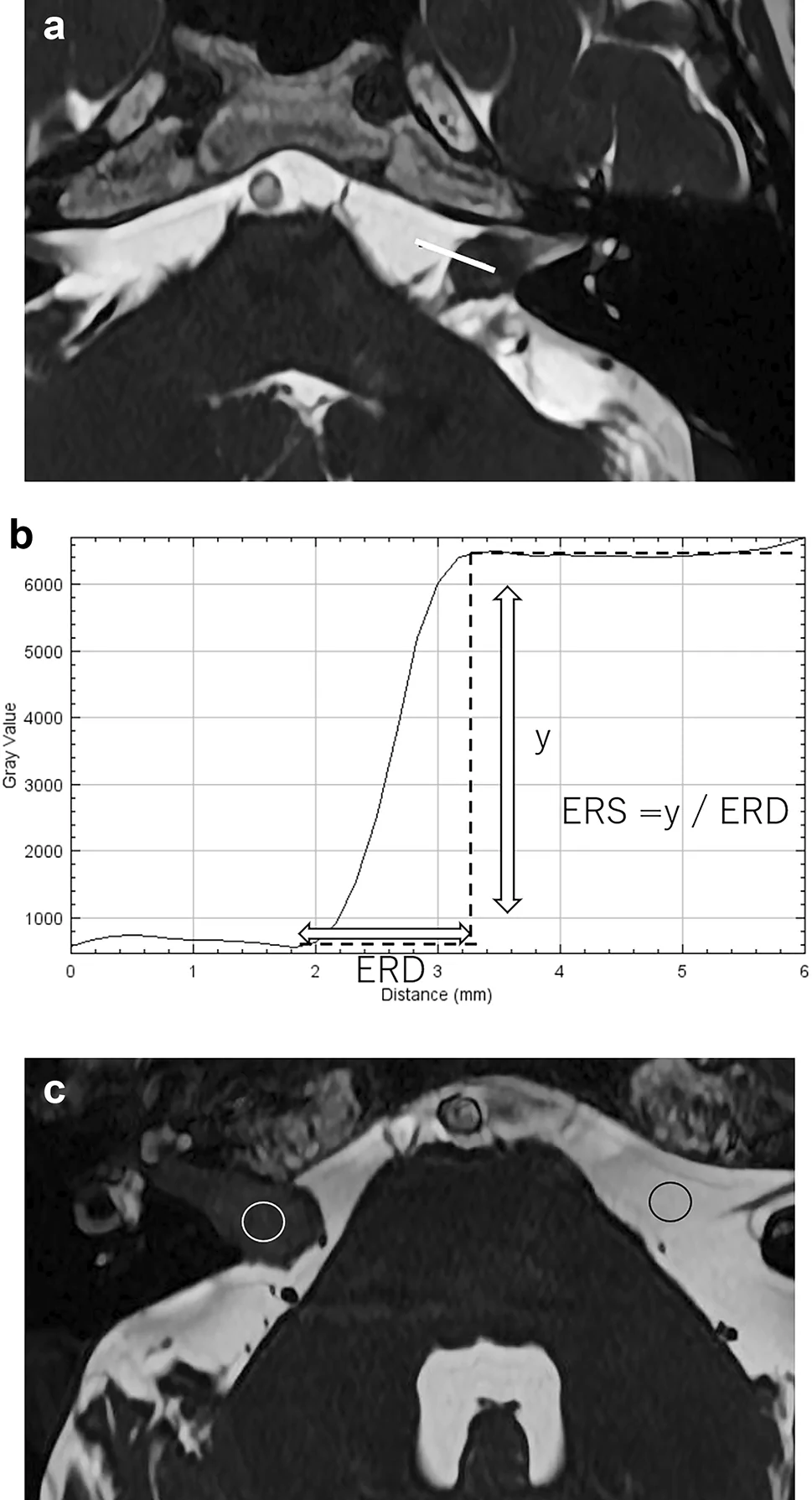
**A** A linear region of interest (ROI [white line]) was drawn perpendicularly across the tumor–cerebrospinal fluid (CSF) boundary from the tumor center toward the CSF. **B** The edge rise distance (ERD) and edge rise slope (= y/ERD) were derived from the signal intensity profile along the ROI. ERD was defined as the linear distance between the points where the signal intensity was 10% and 90% of the signal intensity difference between the maximum value (CSF) and the minimum value (tumor) on the profile. y means the difference insignal intensity between the 10% and 90% levels of the signal intensity profile. **C** Circular or oval ROIs were seton the tumor (white circle) and CSF in the prepontine cistern (black circle)

For the sharpness analysis, a linear region of interest (ROI) was drawn perpendicularly across the tumor–CSF boundary from the tumor center toward the CSF (Fig. 2a). Care was taken to match the ROI locations in the SR-DLR, DLR, and ZIP images. The signal intensity profile along the ROI was recorded, and the ERD and ERS were measured from the signal intensity profile (Fig. 2b). ERD was defined as the linear distance between the points where the signal intensity was 10% and 90% of the signal intensity difference between the maximum value (CSF) and the minimum value (tumor) on the profile.

For the SNR, CNR, SI, and CR analyses, a circular or oval ROI (diameter: 3–5 mm) was placed within both the prepontine cistern CSF and the tumor on axial images (Fig. 2c). As much consistency as possible in ROI placement and size was maintained in the SR-DLR, DLR and ZIP images.

These calculations were performed:

*SNR_TUMOR_ = SI_TUMOR_/SD_TUMOR_,
*SNR_CSF_ = SI_CSF_/SD_CSF_,
*CNR = (SI_CSF_ − SI_TUMOR_)/√((SD_TUMOR_^2^ + SD_CSF_^2^) / 2), and.
*CR = SI_CSF_/SI_TUMOR_

where SI_TUMOR_ is the mean signal intensity of the vestibular schwannoma, SI_CSF_ is the mean signal intensity of the CSF, SD_TUMOR_ is the standard deviation of the tumor signal intensity, and SD_CSF_ is the standard deviation of the CSF signal intensity.

### Statistical analyses

All statistical analyses were performed in the R software environment (version 4.1.2: The R Foundation for Statistical Computing, Vienna, Austria [https://www.r-project.org/]). For the qualitative image analysis, scores for SR-DLR were compared with those for DLR and ZIP using the Wilcoxon signed-rank test separately for each reader. For the quantitative image analysis, SR-DLR was compared with DLR and ZIP using either the paired *t*-test or the Wilcoxon signed-rank test, depending on the distribution of each metric. ERD, SNR, and CNR were normally distributed and were therefore analyzed using paired *t*-tests, whereas ERS, SI_CSF_, SI_TUMOR_, and CR were analyzed using the Wilcoxon signed-rank test. Inter-rater agreement for the qualitative assessments among three readers was evaluated using Kendall’s coefficient of concordance (Kendall’s *W*). The primary pairwise comparisons of interest were SR-DLR versus DLR and SR-DLR versus ZIP; therefore, Bonferroni correction was applied only to these two planned comparisons, and a *p* value of < 0.025 (0.05/2) was considered statistically significant. No additional multiplicity adjustment was applied across readers or across individual endpoints.

## Results

### Patient characteristics

The mean maximum diameter of the tumors in the 39 analyzed patients was 14.1 ± 5.8 mm (range, 3–25 mm), and the side distribution was 19:20 (right: left). No patient had multiple or bilateral tumors. The tumor locations included isolated internal auditory canal involvement (*n* = 11), isolated CPA involvement (*n* = 1), and combined CPA–internal auditory canal involvement (*n* = 27).

### Qualitative image analysis

**Fig. 3:**
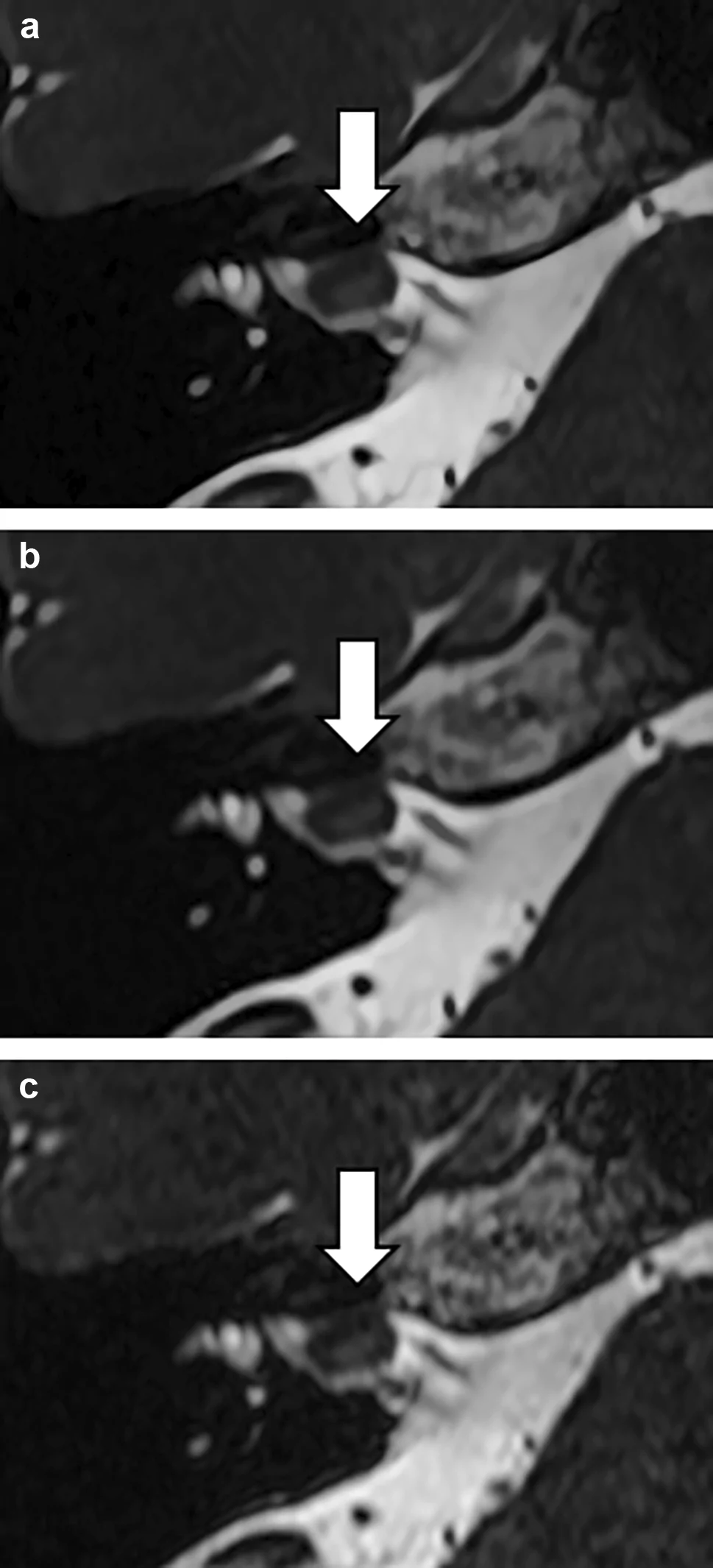
**A**–**C** Three-dimensional constructive interference in steady state brain magnetic resonance images for a 23-year-old woman with a right vestibular schwannoma. The degree of depiction of the vestibular schwannoma (arrows) was rated by readers 1–3 as (A) 4/4/3 in the super-resolution deep learning reconstruction, **B** 4/3/3 in the deep learning reconstruction, and **C** 3/2/2 in the zero-filling interpolation

**Fig. 4:**
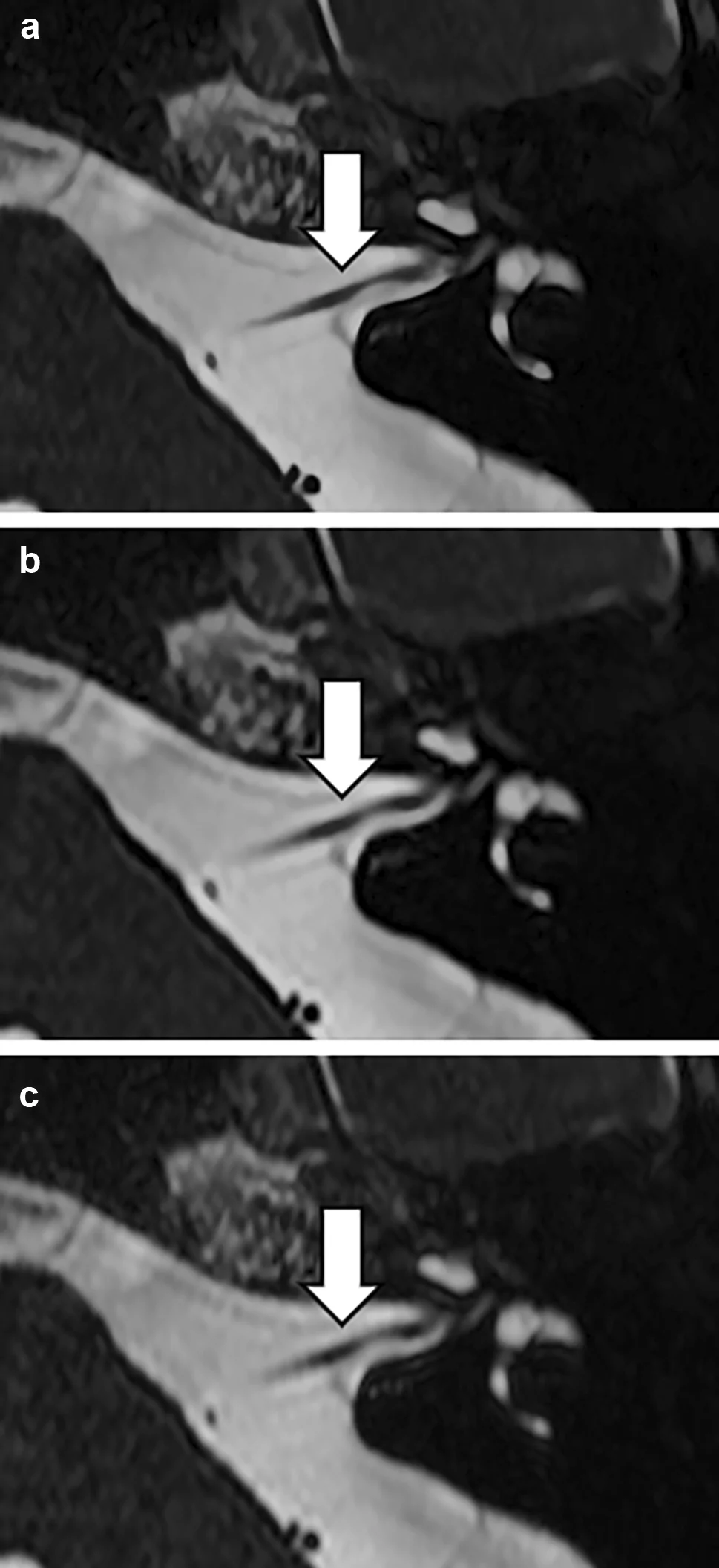
**A**–**C** Three-dimensional constructive interference in steady state brain magnetic resonance images for a 73-year-old woman with a right vestibular schwannoma. The degree of depiction of the contralateral (left) facial (arrows) and vestibulocochlear nerves was rated by readers 1–3 as **A** 4/4/3 in the super-resolution deep learning reconstruction, **B** 4/3/3 in the deep learning reconstruction, and **C** 3/2/2 in the zero-filling interpolation

**Fig. 5:**
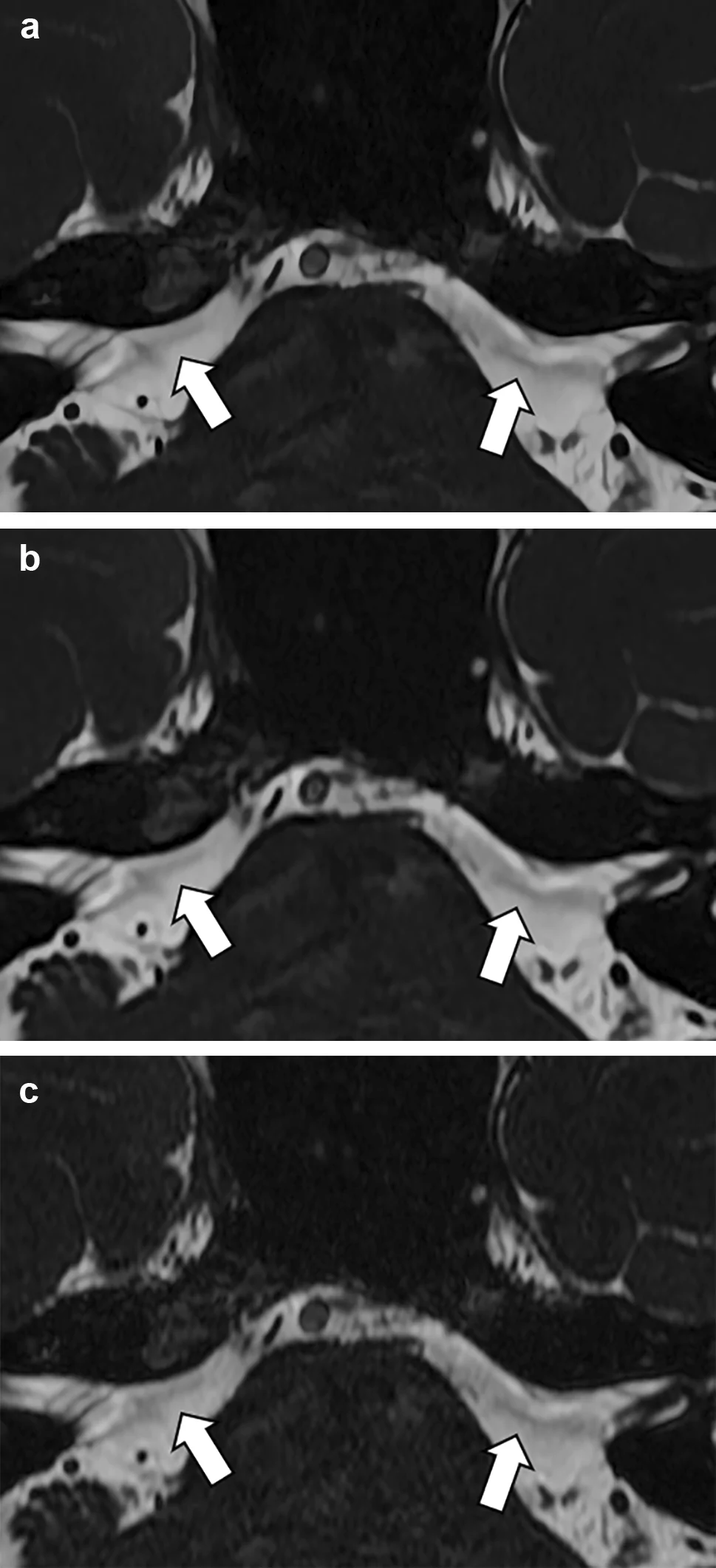
**A**–**C** Three-dimensional constructive interference in steady-state brain magnetic resonance imaging of a 56-year-old man with a right vestibular schwannoma. A band-like area of low signal intensity was observed in the cerebellopontine angle (arrows), which was considered a banding artifact. The overall artifact score was rated 2/2/2 by Readers 1–3 for all three methods: **A** super-resolution deep learning reconstruction, **B** deep learning reconstruction, and **C** zero-filling interpolation

Table 1 summarizes the results of the qualitative image analysis. All readers scored SR-DLR as significantly superior to ZIP in terms of visualization of the vestibular schwannoma (Fig. 3) and facial and vestibulocochlear nerves (Fig. 4) (*p* < 0.001). Reader 2 scored SR-DLR as significantly better than DLR for visualization of the vestibular schwannomas and facial and vestibulocochlear nerves (*p* < 0.001), whereas readers 1 and 3 scored the visualization of both as almost equivalent. All readers scored SR-DLR sharpness as significantly superior to that for both DLR and ZIP (*p* < 0.001). The noise score showed that SR-DLR was significantly superior to ZIP for readers 1 and 2 (*p* < 0.001), while reader 3 rated them as equivalent (*p* = 0.378). Regarding artifacts, SR-DLR did not demonstrate a significant advantage over DLR or ZIP for any reader. SR-DLR diagnostic acceptability was scored higher than that for ZIP (*p* < 0.001). Readers 1 and 2 scored SR-DLR diagnostic acceptability as significantly superior to that for DLR (*p* < 0.001), whereas reader 3 scored the methods as nearly equivalent (*p* = 0.802). Inter-rater agreement among the three readers ranged from fair to good (Kendall’s *W* = 0.345–0.732), with the highest agreement observed in diagnostic acceptability and sharpness. In the evaluated cases, the visually identifiable artifacts were predominantly mild banding artifacts, with occasional susceptibility-related signal irregularities. Marked motion artifacts were uncommon in this cohort, and no reconstruction-specific artifact pattern was consistently observed with SR-DLR, DLR, or ZIP. Representative examples are shown in Fig. 5.

**Table 1:** Qualitative image analysis results

	Reader	Patients (n) by score (5/4/3/2/1 or 4/3/2/1 or 3/2/1)			SR-DLR comparison		Inter-rater agreement
		SR-DLR	DLR	ZIP	vs. DLR	vs. ZIP	Kendall’s *W*
*Depiction of the vestibular schwannoma*							0.643
	1	30/8/1/0	32/6/1/0	2/36/0/1	0.484	< 0.001*	
	2	36/3/0/0	4/31/4/0	0/2/35/2	< 0.001*	< 0.001*	
	3	3/29/7/0	2/30/7/0	1/17/21/0	0.777	< 0.001*	
*Depiction of the facial and vestibulocochlear nerves*							0.595
	1	28/9/2/0	30/7/2/0	9/27/3/0	0.594	< 0.001*	
	2	32/7/0/0	3/27/9/0	0/6/33/0	< 0.001*	< 0.001*	
	3	2/30/7/0	1/33/5/0	0/20/19/0	0.824	< 0.001*	
*Sharpness*							0.729
	1	37/2/0/0	8/31/0/0	0/37/2/0	< 0.001*	< 0.001*	
	2	38/1/0/0	4/33/2/0	0/1/38/0	< 0.001*	< 0.001*	
	3	30/9/0/0	16/22/1/0	2/30/7/0	< 0.001*	< 0.001*	
*Noise*							0.549
	1	36/3/0/0	35/4/0/0	0/32/7/0	0.766	< 0.001*	
	2	38/1/0/0	25/13/1/0	0/6/33/0	0.001*	< 0.001*	
	3	11/20/8/0	13/20/6/0	9/20/10/0	0.267	0.378	
*Artifacts*							0.345
	1	3/36/0	4/35/0	0/39/0	0.773	0.149	
	2	0/38/1	0/39/0	0/38/1	1.000	1.000	
	3	0/39/0	0/39/0	0/38/1	N/A	1.000	
*Diagnostic acceptability*							0.732
	1	22/16/1/0/0	5/32/2/0/0	1/33/5/0/0	< 0.001*	< 0.001*	
	2	31/8/0/0/0	0/25/13/1/0	0/9/30/0/0	< 0.001*	< 0.001*	
	3	5/26/8/0/0	1/33/5/0/0	0/14/23/2/0	0.802	< 0.001*	

*DLR* deep learning reconstruction, *SR-DLR* super-resolution deep learning reconstruction, *ZIP* zero-filling interpolation, *N/A* not applicable

*Statistically significant differences

The numbers of patients with each score (5/4/3/2/1 or 4/3/2/1 or 3/2/1) are shown

### Quantitative image analysis

Table 2 summarizes the results of the quantitative analysis. ERD was significantly reduced, and ERS was significantly increased in SR-DLR images compared with both DLR (*p* < 0.001) and ZIP (*p* < 0.001) images. SR-DLR showed significantly higher SI_CSF_ and significantly lower SI_TUMOR_ compared with ZIP (*p* < 0.001 and *p* = 0.002, respectively). No significant differences in SI_CSF_ and SI_TUMOR_ were found between SR-DLR and DLR (*p* = 0.166 and *p* = 0.058, respectively). CR in SR-DLR images showed significant improvement compared to DLR (*p* = 0.023) and ZIP (*p* < 0.001). SNR_CSF_, SNR_TUMOR_, and CNR were significantly greater in SR-DLR images than in ZIP images (*p* < 0.001), but not statistically significantly different from those in DLR images.

**Table 2:** Quantitative image analysis results

	Mean score			SR-DLR comparison	
	SR-DLR	DLR	ZIP	vs. DLR	vs. ZIP
Vestibular schwannoma ERD (mm)	3.4 ± 1.1	4.0 ± 1.1	4.3 ± 0.9	< 0.001*	< 0.001*
Vestibular schwannoma ERS (mm^− 1^)	2480 ± 1383	1854 ± 896	1415 ± 425	< 0.001*	< 0.001*
SNR_CSF_	34.6 ± 10.9	33.7 ± 11.5	23.4 ± 4.7	0.472	< 0.001*
SNR_TUMOR_	6.2 ± 2.5	6.2 ± 2.3	4.2 ± 1.0	0.916	< 0.001*
CNR	29.5 ± 9.6	29.2 ± 9.7	20.5 ± 4.5	0.573	< 0.001*
SI_CSF_	6307 ± 1202	6138 ± 1143	6131 ± 1245	0.166	< 0.001*
SI_TUMOR_	942 ± 333	947 ± 310	967 ± 300	0.058	0.002*
CR	7.3 ± 2.3	7.0 ± 2.1	6.7 ± 1.6	0.023*	< 0.001*

*SR-DLR* super-resolution deep learning reconstruction, *DLR* deep learning reconstruction, *ZIP* zero-filling interpolation

*Statistically significant differences

Data are presented as mean ± standard deviation. Although ERS, SI_CSF_, SI_TUMOR_, and CR were analyzed using the Wilcoxon signed-rank test due to their non-normal distributions, their values are expressed as mean ± standard deviation for descriptive purposes and consistency with other metrics. CNR, contrast-to-noise ratio; CR, contrast ratio; ERD, edge rise distance; ERS, edge rise slope; SI_CSF_, signal intensity of the cerebrospinal fluid; SI_TUMOR_, signal intensity of the vestibular schwannoma; SNR_CSF_, signal-to-noise ratio for cerebrospinal fluid; SNR_TUMOR_, signal-to-noise ratio for the vestibular schwannoma

## Discussion

In this study, image quality on 3D-CISS imaging was superior with SR-DLR reconstruction to that with ZIP reconstruction for the evaluation of vestibular schwannomas. Sharpness was also better for SR-DLR than for DLR reconstruction, as reflected in a reduced ERD, higher ERS, and better diagnostic acceptability scores, while artifact scores were comparable across methods and noise performance remained similar overall.

The qualitative analysis by each reader revealed that SR-DLR was significantly better than ZIP in depicting vestibular schwannomas and facial and vestibulocochlear nerves (*p* < 0.001). One reader (reader 2) scored SR-DLR images as significantly improved compared with DLR images, whereas two readers (reader 1 and 3) scored them as approximately equivalent. All readers scored SR-DLR as significantly better than either DLR or ZIP in sharpness (*p* < 0.001). The foregoing results suggest that the visibility of small anatomic structures important for evaluation is improved with SR-DLR. A previous study also showed that, in the CPA regions, images from a 3D fast asymmetric spin echo sequence reconstructed using DLR outperformed those not using DLR in terms of depicting tumors, cranial nerves, vessels, and spatial relationships [18]. Reconstructions of 3D-CISS imaging also showed a similar trend, supporting the findings of the present study. To the best of our knowledge, our study is the first to demonstrate the usefulness of 3D-CISS imaging reconstructed using SR-DLR and DLR for evaluating vestibular schwannomas in the CPA region.

Quantitative analysis supports the qualitative results. ERD was significantly reduced in SR-DLR compared with both DLR and ZIP reconstructions, and ERS was significantly increased in SR-DLR compared with both DLR and ZIP reconstructions. Conventionally, improving the sharpness of MR images involves reducing the voxel size. While increasing the ZIP interpolation coefficient can artificially enhance image granularity, it often introduces ringing artifacts (Gibbs phenomenon) and blurring in standard reconstruction. SR-DLR is a new strategy to improve MR image sharpness without increasing noise and artifacts [14, 19, 20]. Unlike conventional ZIP, SR-DLR utilizes neural networks to suppress artifacts even when high interpolation coefficients are applied, effectively achieving both high spatial resolution and low noise.

With respect to noise, the SNR and CNR are significantly higher with SR-DLR than with ZIP reconstruction. Even in subjective evaluations, two readers with more years of experience (reader 1 and 2) rated it as having significantly less noise with SR-DLR than with ZIP. However, the slight improvement in SNR and CNR with SR-DLR compared with DLR reconstruction was nonsignificant. In the subjective evaluation, one reader (reader 2) rated it as having significantly less noise, while two readers (reader 1 and 3) rated it as having no significant difference between SR-DLR and DLR. This suggests that SR-DLR reconstruction maintains comparable noise performance while improving structural clarity. Those results might reflect the improved spatial resolution achieved with SR-DLR, confirming that the improvement in sharpness is evident both subjectively and objectively, in accord with a previous report [14]. In this study, SR-DLR significantly improved CR without increasing noise, as measured by the standard deviation within the ROI. While CNR is generally an important indicator of lesion visibility, our findings suggest that, in 3D-CISS imaging, the benefit of SR-DLR may be related more to improved contrast between the vestibular schwannoma and CSF than to noise reduction alone. Previous studies have shown that the effects of deep-learning-based reconstruction on signal intensity and contrast are not uniform across MRI applications and may vary according to the sequence and anatomical region [21, 22]. In our study, the CR increase was accompanied by a higher SI_CSF_ and a lower SI_TUMOR_, but the mechanism underlying these changes remains uncertain. Because the reconstruction algorithm is proprietary and the internal decision process of deep learning–based reconstruction is not directly interpretable, our results do not allow a definitive explanation for the observed signal intensity changes. Importantly, CR in this study was derived from separate ROIs placed within the vestibular schwannoma and the CSF, rather than from direct measurements at the tumor–CSF boundary. Therefore, our data do not allow us to determine whether SR-DLR would similarly increase, preserve, or reduce contrast in other tissue combinations. The observed CR improvement should thus be interpreted as a sequence- and tissue-combination-specific finding rather than as evidence of a universal contrast-enhancing effect of SR-DLR. Artifact scores for SR-DLR were comparable to those for conventional DLR and ZIP, with no statistically significant differences. Because artifacts were assessed in this study using a single overall artifact score rather than separate evaluations of individual artifact types, our data do not allow detailed discussion of the behavior of specific artifacts. The visually identifiable artifacts in our cohort were predominantly mild banding artifacts with occasional susceptibility-related signal irregularities (Fig. 5), whereas marked motion artifacts were uncommon. Accordingly, our results indicate only that SR-DLR did not clearly worsen the overall artifact burden in 3D-CISS imaging; they do not demonstrate a specific advantage in artifact suppression. Further studies designed for artifact-specific analysis would be required to clarify the effects of SR-DLR on individual artifact types. The enhanced sharpness is particularly valuable for differentiating the delicate course of the facial and vestibulocochlear nerves within the cistern—a clinical task where standard ZIP often fails due to blurring. In the overall assessment of diagnostic acceptability, readers 1 and 2 scored SR-DLR images significantly higher than DLR images (both *p* < 0.001), whereas reader 3 found no significant difference (*p* = 0.802). That variation might be a result of the lower experience level of reader 3, who had only one year of experience, compared with the six and three years of experience for readers 1 and 2. In addition to sharper structural definition, improved diagnostic acceptability of overall image quality may facilitate easier preoperative assessment of the relative positions of the tumors, facial and vestibulocochlear nerves. Furthermore, in post-treatment cases, it may become easier to detect minute changes in tumors.

This study has several limitations. First, the relatively small sample size might limit the generalizability of our results. Future prospective studies with larger and more diverse populations are needed to further validate the clinical utility of SR-DLR. Second, this study compared SR-DLR images with DLR/ZIP images, which are reconstructed using different matrix sizes and pixel spacings. Therefore, the observed improvement in image quality may include not only the effect of the SR-DLR algorithm itself but also the increased sampling density associated with higher matrix sizes (the sampling benefit). Accordingly, the present results should be interpreted as reflecting the overall performance of the implemented reconstruction setting rather than the isolated algorithmic effect alone. Third, SR-DLR reconstruction improves only the in-plane spatial resolution and not the through-plane resolution; reformatted multiplanar reconstruction images might therefore not reach the same degree of improvement observed in axial source images. Fourth, the multiple comparison correction in this study was limited to the two primary comparisons of interest. We did not strictly adjust the significance level across all readers and evaluation items, treating them as independent observations. This approach might be less conservative than an all-encompassing correction, and the results should be interpreted as exploratory for certain sub-metrics. Fifth, we did not evaluate the inter-observer reproducibility of the quantitative measurements. Because metrics such as ERD, ERS, SNR, and CNR depend on manual ROI placement and edge-profile processing, the results may have been influenced by inter-observer variability. Given this potential sensitivity, these quantitative findings should be interpreted as exploratory. Further studies involving multiple observers and larger patient cohorts are necessary to confirm the robustness and reproducibility of these quantitative advantages of SR-DLR. Finally, although high-resolution 3D-CISS imaging is expected to facilitate the detection of small cranial nerve tumors, we did not evaluate detection performance because the tumors in our patient sample were relatively large, averaging approximately 14 mm. According to a previous report, the average size of vestibular schwannomas when detected is 10 mm [4]. Future studies should investigate the detection capability for small tumors.

## Conclusion

This study demonstrated that SR-DLR improved 3D-CISS imaging quality for evaluating vestibular schwannomas and adjacent cranial nerves compared with ZIP reconstruction, resulting in higher diagnostic acceptability among more experienced readers. Compared with conventional DLR, SR-DLR provided clearer images with better sharpness, while maintaining similar noise and artifact levels. These findings support SR-DLR as a promising tool for visualizing small neural structures.

## Data Availability

Due to the nature of this research, patients of this study did not agree for their data to be shared publicly, so supporting data is not available.
